# Immature neutrophils in cord blood exert increased expression of genes associated with antimicrobial function

**DOI:** 10.3389/fimmu.2024.1368624

**Published:** 2024-03-26

**Authors:** Eliška Miková, Viktor Černý, Olga Novotná, Petra Petrásková, Kristýna Boráková, Zdenek Hel, Jiří Hrdý

**Affiliations:** ^1^ Institute of Immunology and Microbiology, First Faculty of Medicine, Charles University, Prague, Czechia; ^2^ Department of Neonatology, Institute for the Care of Mother and Child, Prague, Czechia; ^3^ Pathology Department, University of Alabama at Birmingham, Birmingham, AL, United States

**Keywords:** neutrophils, cord blood, myeloperoxidase, defensins, oxidative burst

## Abstract

**Introduction:**

The immune systems of both the mother and the newborn face significant challenges during birth. Proper immune regulation after birth is essential for the survival of neonates. Numerous studies have demonstrated that the neonatal immune system is relatively immature, particularly in its adaptive arm, placing the primary responsibility for immune surveillance on innate immunity.

**Methods:**

Given the significant role of neutrophils in protecting the neonate after birth, we conducted a study investigating the properties of neutrophils in newborn cord blood using various methodological approaches.

**Results:**

Our findings demonstrate the presence of immature low-density neutrophils in the cord blood, which are likely responsible for the observed elevated expression of genes coding for proteins essential to antimicrobial response, including myeloperoxidase, neutrophils elastase, and defensins.

**Discussion:**

We propose that these cells function normally and support the protection of newborns early after birth. Furthermore, our results suggest that the mode of delivery might significantly influence the programming of neutrophil function. The presented findings emphasize the importance of distinct neutrophil subpopulations in neonatal immunity and their potential impact on early postnatal health.

## 1 Introduction

The neonatal immune system is characterized by its immaturity and generally lower functional capacity compared to the adult immune system. Neonatal adaptive immune responses are impaired since exposure to antigens *in utero* is limited (1). In the early days after birth, the newborn is primarily protected by antibodies of maternal origin, delivered either prenatally or postnatally through breastfeeding. The innate immune system, with neutrophils in the first line of defense, plays a crucial role in cellular immunity against potentially harmful pathogens in the early stages of infection. Despite this, due to the deficiency in immune functions, newborns are at a higher risk of neonatal sepsis, with preterm children being particularly vulnerable (2).

Interestingly, neonates delivered by caesarean section have more pronounced immaturity of the immune system, further increasing the risk of a severe course of infection. It is well-established that the mode of delivery plays a crucial role in the future health of the newborn, both in the early postnatal period and in the long term. For example, there is a higher incidence of childhood asthma (3) and inflammatory bowel disease (4, 5) in individuals born via caesarean section. Additionally, the delivery mode can significantly influence neutrophil functional response, namely reactive oxygen species (ROS) production (6) and migration (7) were increased in cord blood neutrophils of neonates delivered vaginally.

For a long time, neutrophils were considered to be a short-lived homogeneous cell population with functions limited to their suicidal nature at the site of inflammation. In recent years, the view of neutrophil biology has changed extensively. It was shown that neutrophils can live up to five days (8). Although this observation was challenged (9) it seems that the uncertainty about the length of neutrophil survival mainly stems from the limitation of techniques and methodological approaches used to evaluate neutrophil half-life *in vivo* (10). Neutrophils stably replenish their numbers in blood by exchange of mature neutrophils from the bone marrow and can influence the adaptive immune system either directly (11–13) or via the production of cytokines (14, 15). Neutrophils are now considered a heterogeneous population with an extensive range of functions (16–18).

Although neutrophil subpopulations may be distinguished based on multiple criteria including the presence of transcription factors (19), the cell separation method based on centrifugation in density gradient is most frequently employed. Neutrophils sedimenting in the erythrocyte fraction have been termed high-density neutrophils (HDN) and are typically considered “classical” pro-inflammatory neutrophils. Low-density neutrophils (LDN) in the peripheral blood mononuclear cells (PBMC) fraction were initially described in 1986 in patients with autoimmune disease (20). Since then, LDNs were found in increased numbers in various inflammatory conditions, including cancer, cardiovascular diseases, autoimmune diseases, and infections, suggesting their potential roles in inflammation (21–24). Hassani et al. (25) showed that LDN expand in patients with chronic inflammation and, following induction with LPS, they consist primarily of CD16^dim^CD62L^high^ neutrophils (25). LDN were shown to exert suppressive function during carcinogenesis (26) and pregnancy (27–29). Interestingly, LDN can exert pro-inflammatory function under specific circumstances, for example in patients with systemic lupus erythematosus (SLE) (16, 30) (for a detailed review of this topic see (31)). Villanueva and colleagues (32) showed an enhanced expression of proteins linked to neutrophil antimicrobial activity in systemic lupus erythematosus (SLE) [e.g., myeloperoxidase (MPO), neutrophil elastase (ELANE), alpha defensin 4 (DEFA4)].

However, the functional properties of LDNs remain contradictory. Different studies have reported conflicting findings, with some suggesting LDNs have T cell activating properties (30, 33) while others argue for suppressive effects (34–36). Similarly, LDNs have been linked to both pro-tumorigenic and anti-tumorigenic activities, pro-inflammatory and anti-inflammatory qualities, as well as variable phagocytic capacities and ROS production compared to normal-density neutrophils (37–39).

Previously, it was shown that neutrophils in cord blood display immature phenotype with impaired antimicrobial functions; however, the reported studies show low degree of consistency. Ssemaganda et al. (40) found that in cord blood (CB), LDN represented the most significant part of CD15^+^ neutrophils compared to peripheral blood (PB) of mothers and expressed low levels of CD16, suggesting their relative immaturity. A similar phenotype (low expression of CD16 with high expression of CD64) was observed in CB of children with neonatal sepsis (41). This group suggested that CB LDN are phenotypically distinct from adult LDN.

In this study, we investigate the phenotype and functional properties of neutrophils in umbilical cord blood, which can significantly impact the neonate’s early days after delivery and overall well-being. Our findings confirmed the presence of immature CD16^low^ neutrophil subpopulations in umbilical cord blood. This population may be responsible for increased expression of genes associated with antimicrobial response, including defensins and myeloperoxidase. Interestingly, upon challenge with an inflammatory stimulus (*Escherichia coli*), both the cord blood and maternal peripheral blood neutrophils displayed normal levels of activation and oxidative burst.

## 2 Materials and methods

### 2.1 Sample collection

This study was approved by the Ethical Committee at the Institute for the Care of Mother and Child in Podolí, Prague, Czech Republic. All mothers and healthy volunteers signed a written informed consent before sample collection. Cord and peripheral blood were collected at the Institute for the Care of Mother and Child in Prague, the Czech Republic. Umbilical cord blood (20-30 ml) was drawn from the umbilical vein with a sterile needle into a 50 ml flask with heparin (10U/ml). During the standard pre-labour examination, maternal peripheral blood (2-5 ml) was drawn into K_2_EDTA or heparin-containing tubes. Blood samples were stored at RT in a dark box until pick-up. Peripheral blood (2-5 ml) of healthy women volunteers was drawn into K_2_EDTA or heparin-containing tubes. Details of the studied cohort of mothers and children are listed in **Table 1**.

**Table 1:** The details of the studied cohort.

Number of women participating in this study		59
		Mean ± SD
Age (years)		34 ± 3.7
Week of pregnancy		38.7 ± 0.7 + 2.1 ± 1.9
Delivery	Caesarean section	41
	Vaginal delivery	18
Gender of the child	Female	25
	Male	34
Weight (g)		3,324.7 ± 383.8
Length (cm)		49.3 ± 1.7
Apgar score	1min	9.8 ± 0.3
	5min	9.9 ± 0.1
	10min	10 ± 0.1

### 2.2 Flow cytometry analysis

Whole blood was stained with anti-human CD14 (clone 61D3, Invitrogen, USA), CD15 (clone HI98, BioLegend, USA), CD16 (clone CB16, Invitrogen), CD62L (clone DREG-56, BioLegend, USA), and CD64 (clone 10.6, BioLegend, USA). After 15 min incubation in the dark, erythrocytes were lysed with 10x diluted Lysis Buffer (QIAGEN, Netherlands), and samples were washed twice with PBS. Samples were measured on BD FACS CantoTM II Flow Cytometer (BD Biosciences, USA) equipped with the BD FACS Diva Software v6.1.2 and analyzed with FlowJo v10.10.0. (FlowJo LLC, USA) software. Gating strategy is shown in **Supplementary Figures 1**, **2**.

### 2.3 Cell isolation

For the isolation of the whole neutrophil population, the EasySepTM Direct Human Neutrophil Isolation Kit (STEMCELL Technologies, Canada) was used. Neutrophils were isolated according to the manufacturer’s instructions with slight modifications to increase cell yield and purity. In the first step, 50 μl of 0.5 M EDTA/1 ml blood was added, and the first incubation was extended to 6 min. Isolated cells were washed once with PBS and counted. Cell viability and purity were assessed by Trypan blue and flow cytometry, respectively. The purity of the isolated neutrophil population was higher than >96%. Mononuclear cell fraction was obtained as follows. The whole cord or peripheral blood was diluted 1:1 with PBS and layered carefully over Ficoll-PaqueTM PLUS (density 1.077 g/l) in a conical tube. Density-gradient centrifugation was performed at 500 x g for 30 min. After centrifugation, blood was separated into CBMC or PBMC-rich layer and granulocyte-rich erythrocyte pellet. Cells from both fractions were washed twice with PBS and counted. LDN were isolated from CBMC using EasySepTM Human CD15 Positive Selection Kit (STEMCELL Technologies, USA) according to the manufacturer’s instructions. HDN were isolated using EasySepTM Direct Human Neutrophil Isolation Kit (STEMCELL Technologies, USA) from the erythrocyte fraction as described above.

### 2.4 Quantitative PCR

Total RNA from isolated neutrophils was extracted using the RNeasy® Mini Kit (QIAGEN, Netherlands) according to the manufacturer’s instructions with the addition of the optional RNase-Free DNase I (QIAGEN) incubation step to prevent genomic DNA contamination. The purity and concentration of the RNA were determined using NanoDrop 1000 Spectrophotometer (Thermo Scientific, USA). Total RNA was reverse transcribed to cDNA using the High Capacity cDNA Reverse Transcription Kit (Applied Biosystems) according to the manufacturer’s instructions. The relative expression of selected genes was performed using the Luna® Universal qPCR Master Mix (New England BioLabs, UK) and TaqMan® probes (Applied Biosystems, USA) and measured on the LightCycler® 480 II Real-Time PCR System (Roche Molecular Systems, USA). All tested genes and their respective probe assay IDs are listed in **Table 2**.

**Table 2:** List of primers and probes used of analyses of genes of interest.

Gene symbol	Assay ID	Gene name
ACTB	Hs99999903_m1	Actin beta
ARG1	Hs00163660_m1	Arginase 1
CD274	Hs00204257_m1	CD274 molecule (PD-L1)
DEFA3	Hs00414018_m1	Defensin alpha 3
DEFA4	Hs00157252_m1	Defensin alpha 4
ELANE	Hs00357734_m1	Elastase, neutrophil expressed
IDO1	Hs00984148_m1	Indoleamine 2,3-dioxygenase 1
IL10	Hs00174086_m1	Interleukin 10
LTF	Hs00914334_m1	Lactotransferrin
MMP9	Hs00957562_m1	Matrix metallopeptidase 9
MPO	Hs00165162_m1	Myeloperoxidase
PDCD1	Hs00228839_m1	Programmed cell death 1 (PD-1/CD279)
TNF	Hs00174128_m1	Tumor necrosis factor

### 2.5 Myeloperoxidase activation assay

The phagocytic capacity of neutrophils quantified as relative myeloperoxidase (MPO) activity was tested by the FagoFlowExKit (EXBIO, CZ) according to the manufacturer’s instructions with the addition of anti-human anti-CD15 antibody (clone HI98, BD Biosciences) for precise determination of neutrophils. The method utilizes the oxidation of dihydrorhodamine (DHR) 123 to fluorescent rhodamine 123 by activated MPO. The fluorescence was measured using BD FACS CantoTM II Flow Cytometer (BD Biosciences, USA) equipped with the BD FACS Diva Software v6.1.2 and analyzed with FlowJo 7.6.5. (FlowJo LLC, USA) software. The gating strategy and representative histograms are shown in **Supplementary Figure 3**. The relative MPO activity was calculated based on mean fluorescent intensity (MFI) as follows:


$$Relative\;MPO\;activity=\;\frac{MFI_{S}-MFI_{N}}{MFI_{P}-MFI_{N}}\;$$

(MFI_S_ – MFI of sample stimulated with *E. coli*, MFI_P_ – MFI of positive control, MFI_N_ – MFI of negative control) to account for the background fluorescence.

### 2.6 Data analysis and statistical analysis

Flow cytometry results were standardized using Cytometer Setup and Tracking beads (BD Biosciences, USA) and analyzed by FlowJo software using appropriate compensation controls (single staining, compensation beads, isotype control) and fluorescence minus one (FMO) control to set proper gating strategy. Transcript expression of the target gene was quantified relative to endogenous control (housekeeping gene) actin beta (*ACTB*) mRNA levels using the 2^-ΔΔCt^ method as described earlier (42). For flow cytometry analysis and qPCR analysis, parametric statistical tests (unpaired Students *t*-test) were utilized. Acquired data were statistically evaluated and graphically processed using GraphPad Prism 8 (GraphPad Software Inc.) software. Pearson correlation test was employed for the correlation of gene expression with neutrophil subpopulation. Statistically significant differences among the groups were considered when p-values were lower than 0.05.

## 3 Results

### 3.1 Umbilical cord blood has an increased number of CD16^low^ neutrophils.

**Figure 1:**
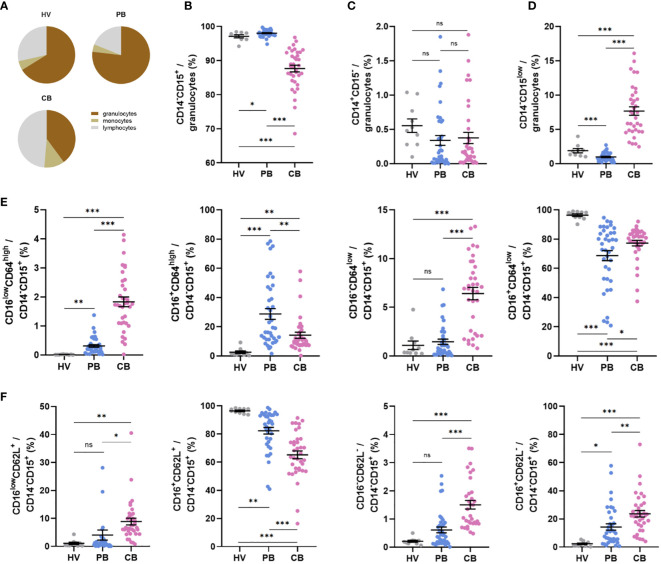
Expression of neutrophil cell surface markers in peripheral blood of healthy women volunteers (HV), maternal peripheral blood (PB), and umbilical cord blood (CB). **(A)** Relative proportion of granulocytes, monocytes, and lymphocytes. **(B)** The relative frequency of neutrophils, gated as CD14^-^CD15^+^. **(C)** The relative frequency of monocytes gated as CD14^+^CD15^-^ cells. **(D)** Relative frequency of CD14^-^CD15^low^ neutrophils. **(E)** Relative frequencies of CD16^low^CD64^high^, CD16^+^CD64^high^, CD16^-^CD64^low^, and CD16^+^CD64^low^ cells within CD14^-^CD15^+^ neutrophils. **(F)** Relative frequencies of CD16^low^CD62L^+^, CD16^+^CD62L^+^, CD16^low^CD62L^-^ and CD16^+^CD62L^-^ within CD14^-^CD15^+^ neutrophils. HV: n = 10, PB: n = 38, CB: n = 36. The data are shown as the mean ± SEM for the whole group. Asterisks indicate *p< 0.05, **p< 0.01, and ***p< 0.001, two-tailed unpaired Student’s *t* test. non-significant (ns) ^ns^p > 0.05.

**Figure 2:**
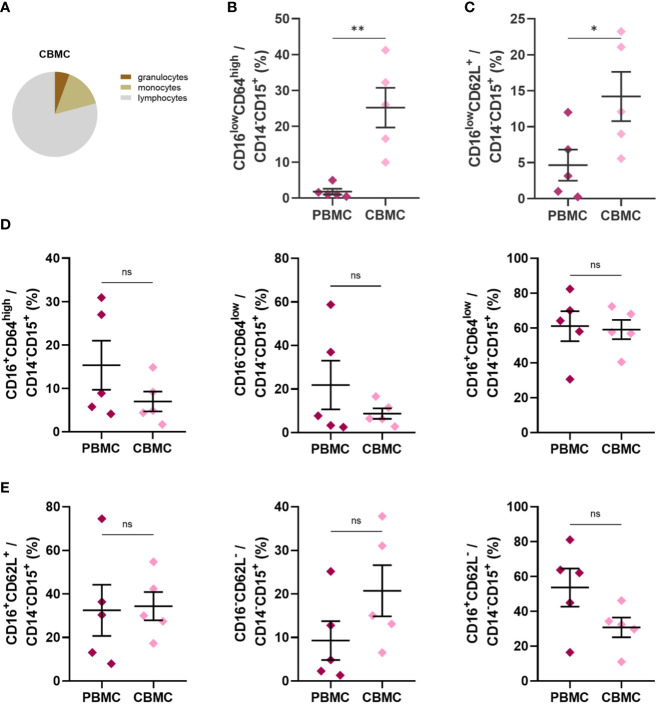
Expression of neutrophil cell surface markers in PB and CB mononuclear cell fraction after density gradient centrifugation. **(A)** Relative proportion of granulocytes, monocytes and lymphocytes. **(B)** CD16^low^CD64^high^CD14^-^CD15^+^ **(C)** CD16^low^CD64^+^CD14^-^CD15^+^ **(D)** Relative frequencies of CD16^+^CD64^high^CD14^-^CD15^+^, CD16^-^CD64^low^CD14^-^CD15^+^ and CD16^+^CD64^low^CD14^-^CD15^+^ **(E)** Relative frequencies of CD16^+^CD62L^+^CD14^-^CD15^+^, CD16^-^CD62L^-^CD14^-^CD15^+^ and CD16^+^CD62L^-^CD14^-^CD15^+^ (PBMC: n = 5, CBMC n = 5). The data are shown as the mean ± SEM for the whole group. Asterisks indicate *p< 0.05, **p< 0.01, two-tailed unpaired Student’s *t* test. non-significant (ns) ^ns^p > 0.05.

Based on previous reports indicating the presence of immature neutrophils in CB (40, 41), we performed phenotype analyses of selected neutrophil cell surface markers reflecting neutrophil maturational and activation status. Neutrophils in CB accounted for only about 40% of total cells compared with proportion of neutrophils in HV (68%) and PB (76%) (**Figure 1A**), whereas their frequency was only slightly elevated in the PB of mothers compared with HV (**Figure 1A**). The frequency of neutrophils identified as CD14^-^CD15^+^ granulocytes (43, 44) was lower in CB (**Figure 1B**), mainly due to the enrichment in CD14^-^CD15^low^ granulocytes (**Figure 1D**), a granulocyte population not previously described (gating strategy in **Supplementary Figures 1**, **2**). Monocytes, identified as CD14^+^CD15^-^ cells (45), did not differ between the study groups (**Figure 1C**). Neutrophils were further gated based on the expression of CD16 (FcγRIIIB) and CD64 (FcγRI), markers considered to reflect neutrophil maturity, and CD62L (L-selectin). As previously reported (41), CD16^low^CD64^high^ neutrophils were increased in CB (**Figure 1E**), with a similar trend observed in the case of CD16^low^CD62L^+^ (**Figure 1F**). Most of the neutrophils in the control group exhibited CD16^+^CD64^low^ and CD16^+^CD62L^+^ phenotype (**Figures 1E, F**), suggesting a mature non-activated neutrophil population. Neutrophils in PB of mothers displayed increased expression of CD64 (**Figure 1E**), suggesting neutrophil activation during pregnancy. To address whether CD16^low^ neutrophils have a low-density characteristics, identical staining and gating strategies were applied to isolated CBMC or PBMC. An increase in the frequency of CD16^low^ neutrophils in CBMC was observed (**Figures 2B, C**), more prominent when cells were gated as CD16^low^CD64^high^ (**Figure 2B**). The other subpopulations did not differ between the experimental groups (**Figures 2D, E**), confirming the nature of CD16^low^ neutrophils as LDN. To better identify the particular subpopulation based on the cell surface presence of CD16, alternative gating was performed (see **Supplementary Figure 2**) and three neutrophil subsets were identified according to CD16 (i.e. CD16 negative (CD16^-^); CD16 dim (CD16^int^) and CD16 bright (CD16^+^)). Proportion of particular neutrophil subsets based on CD16 distribution is presented in **Supplementary Figure 4**. This alternative gating provide us with remarkably elevated proportion of CD16^-^CD64^high^ neutrophils within CD14^-^CD15^+^ cells in CB compared with HV. CD16^int^CD64^high^ neutrophils within CD14^-^CD15^+^ cells were significantly increased in CB compared with HV. CD16^+^CD64^high^ neutrophils were the most abundant in PB in comparison with HV and CB. Proportion of CD16^+^CD64^high^ neutrophils was elevated in CB compared with HV. No difference was observed in proportion of CD16^-^CD64^low^ among HV, PB and CB. On contrary, CD16^+^CD64^low^ were significantly increased in HV compared to both PB and CB. However, CD16^+^CD64^low^ was significantly elevated in CB compared to PB, **Supplementary Figure 4A**. No difference in proportion of CD16^-^CD62L^+^ and CD16^int^CD62L^+^ among HV, PB and CB has been detected. Subset of CD16^+^CD62L^+^ was significantly higher in HV compared to CB. CD16^-^CD62L^-^ were significantly increased in CB compared with HV, **Supplementary Figure 4B**.

### 3.2 Cord blood neutrophils display elevated expression of genes associated with antimicrobial function

**Figure 3:**
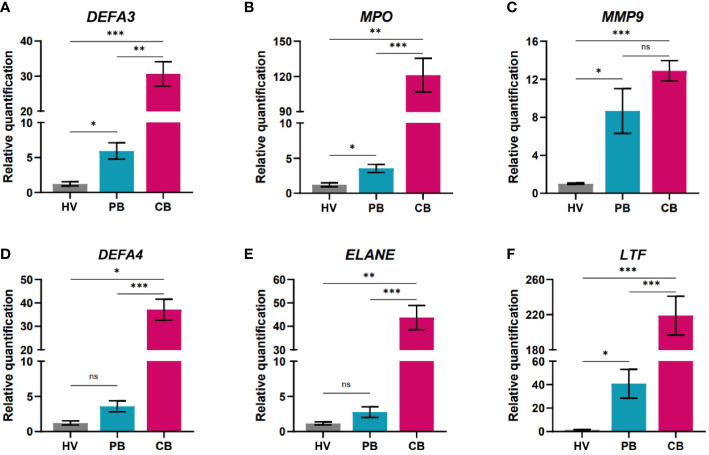
Quantification of gene expression in neutrophils isolated from peripheral blood of healthy women volunteers (HV), maternal peripheral blood (PB), and umbilical cord blood (CB). **(A)** alpha defensin 3 **(B)** myeloperoxidase **(C)** matrix metalloproteinase 9 **(D)** alpha defensin 4 **(E)** neutrophil elastase **(F)** lactotransferrin (HV: n = 6, PB: n = 16, CB: = 54). The data are shown as the mean ± SEM for the whole group. Asterisks indicate *p< 0.05, **p< 0.01, and ***p< 0.001, two-tailed unpaired Student’s *t* test. non-significant (ns) ^ns^p > 0.05.

**Figure 4:**
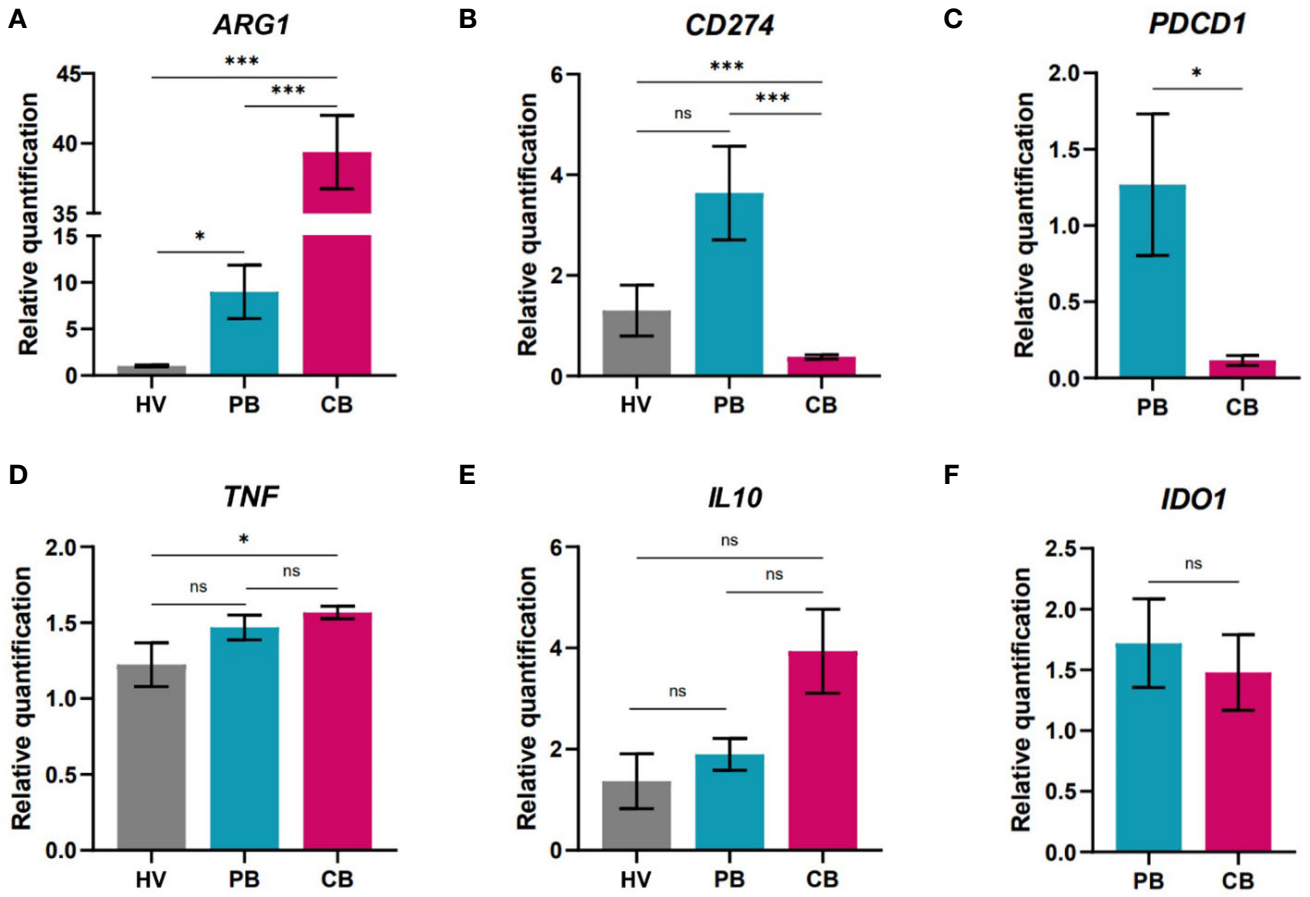
Gene expression analysis of neutrophils isolated from peripheral blood of healthy women volunteers (HV), maternal peripheral blood (PB), and umbilical cord blood (CB). **(A)** arginase **(B)** Programmed death-ligand 1 (PD-L1/CD274) **(C)** Programmed cell death 1 (PB: n = 4, CB: n = 4) **(D)** tumor necrosis factor alpha **(E)** interleukin 10 **(F)** indol amine 2,3 dioxygenase (PB: n = 10, CB = 11). (A, B, D, E HV: n = 6, PB = 9, CB = 17). The data are shown as the mean ± SEM for the whole group. Asterisks indicate *p< 0.05, ***p< 0.001, two-tailed unpaired Student’s *t* test. non-significant (ns) ^ns^p > 0.05.

Next, we determined the expression of genes linked to antimicrobial (**Figure 3**) or suppressive (**Figure 4**) functions in all three study groups. The association of human defensin alpha 3 in anti-infectious immunity has been highlighted (46). On the other hand, elevated presence of alpha defensin 3 (*DEFA3*) in vaginal fluid after premature preterm rupture of membranes pointed to neonatal inflammation (47). In our study, significantly increased gene expression of *DEFA3* was observed in cord blood neutrophils compared to neutrophils of adults (both HV and PB). Importantly, PB neutrophils showed promoted gene expression of *DEFA3* compared to HV suggesting increased readiness to fight against infection, **Figure 3A**. The marker linked with the function of neutrophils is myeloperoxidase which stabile levels during pregnancy can be considered as a biomarker of non-complicated course of pregnancy (48). Cord blood neutrophils shows superior gene expression of *MPO* compared to adult neutrophils (both HV and PB). Interestingly, PB neutrophils exerted elevated gene expression of *MPO* compared to HV confirming higher anti-microbial capacity, **Figure 3B**. There was no difference in the expression of *MMP9* between the cord and maternal peripheral blood, possibly explained by the specific increase in the expression of metalloproteases during pregnancy and labour (49). Another peptide with antimicrobial capacity secreted by neutrophils is defensin alpha 4 (*DEFA4*) (50). We have observed significantly increased gene expression of *DEFA4* in cord blood neutrophils compared to HV and PB. Neutrophil elastase (*ELANE*) is a marker of NET formation in health and disease (51). Here, we showed significantly elevated gene expression of *ELANE* in cord blood neutrophils compared to both HV and PB. Lactotransferrin (*LTF*) is playing a pivotal role in inflammatory homeostasis (52). Therefore, we have evaluated gene expression of *LTF* in cord blood neutrophils and confirmed superior expression of *LTF* in cord blood neutrophils compared with HV and PB highlighting prerequisite of cord blood neutrophils exert antimicrobial functions. PB neutrophils poses still elevated gene expression of *LTF* compared to HV neutrophils. In summary, cord blood neutrophils display highly increased expression of genes coding for proteins playing an important role in antimicrobial response (e.g., myeloperoxidase, defensins, and neutrophil elastase) compared to neutrophils from the mothers or healthy volunteers (**Figures 3A, B, D–F**).

Successful pregnancies were associated with neutrophil subpopulations with prone expression of arginase (*ARG1*). Interestingly, in the case of genes associated with immune suppressive properties, there was an increase in the expression of *ARG1* (**Figure 4A**) in CB neutrophils but limited expression of *CD274* encoding PD-L1, that was highly elevated in neutrophils from maternal PB (**Figure 4**). PD-L1 is a marker identified on neutrophils and its expression is associated with undergoing inflammatory process (53, 54). Therefore, it is not surprising that only marginal PD-L1 expression has been detected in cord blood neutrophils of healthy neonates. Along with the increase in *CD274*, maternal neutrophils also displayed elevated expression of *PDCD1* (**Figure 4C**), a gene translated to the receptor (Programmed cell death protein 1, PD-1) for PD-L1, potentially indicating a negative feedback loop exerted in maternal neutrophils. Only slight increase in the expression of tumor necrosis factor (*TNF*) in CB neutrophils was observed in comparison to HV neutrophils (**Figure 4D**), indicating elevated antimicrobial activity. We have focused on gene expression of immunoregulatory markers as well. Interleukin 10 (*IL10*) is well known cytokine with immunoregulatory/immunosuppressive function preventing undesirable immune responses (55). As another marker suggesting potential immunoregulatory capacity of neutrophils was selected indol amine 2,3 dioxygenase (*IDO*) which expression has been shown limited in more reactive cells (56). Surprisingly, no difference was observed in the expression of these genes of interest (**Figures 4E, F**). Next, the expression of genes of interest was compared in cord blood of children delivered either by vaginal delivery (VD) or caesarean section (CS) (**Supplementary Figure 5**). Interestingly, children delivered by CS displayed elevated expression of defensins and myeloperoxidase. In contrast, children delivered by VD displayed higher expression of genes coding for IL-10 and PD-L1 proteins, indicating elevated proinflammatory settings in children born by CS.

### 3.3 Elevated expression of genes associated with antimicrobial function correlates with the frequency of CD16^low^ neutrophils in cord blood

**Figure 5:**
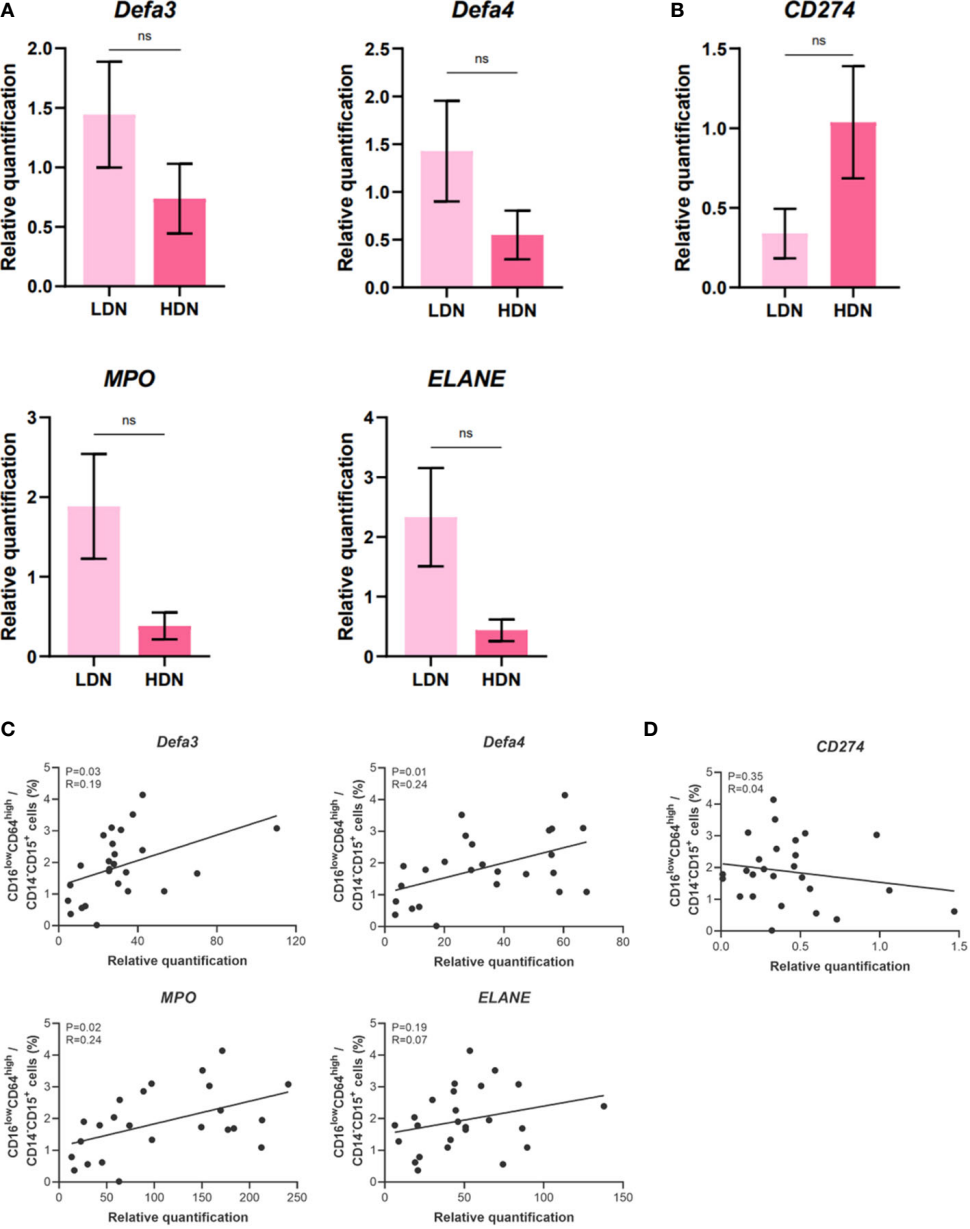
Gene expression analysis of low-density neutrophils (LDN) and high-density neutrophils (HDN) isolated from umbilical cord blood (CB). **(A)** alpha defensin 3, alpha defensin 4, myeloperoxidase, neutrophil elastase. **(B)** Programmed death-ligand 1 (PD-L1/CD274). **(C)** Correlation analysis between the relative gene expression of genes coding for antimicrobial substances and percentage of the neutrophil population (CD14^-^CD15^+^CD16^low^CD64^high^). **(D)** Correlation analysis between the relative gene expression of CD274 and percentage of the neutrophil population (CD14^-^CD15^+^CD16^low^CD64^high^). The data are shown as the mean ± SEM for the whole group. Asterisks indicate ^ns^ *p* > 0.05, two-tailed unpaired Student’s *t* test. Pearson correlation analysis was performed for **(C, D)**. Pearson correlation coefficient and *p*-value were obtained and *p*-value less than 0.05 (*p<* 0.05) was considered statistically significant. non-significant (ns) ^ns^p > 0.05.

Next, we set to elucidate whether the higher relative frequency of LDN in CB might be responsible for the elevated expression of genes associated with antimicrobial function. To this aim, we isolated LDN and HDN from CB and performed gene expression analysis of candidate genes of interest. The data did not reach statistical significance (**Figures 5A, B**), probably due to the low number of samples. Only non-significant tendency toward higher expression of genes associated with antimicrobial response was observed in LDN (**Figure 5A**). In order to overcome the difficulties associated with cell isolation, correlation analysis was performed. The frequency of CD16^low^CD64^high^ neutrophils in CB was correlated with the relative gene expression of examined genes in neutrophils isolated from whole CB (**Figures 5C, D**). Despite quite low value of correlation coefficient, the correlation reaches significance in three of the candidate genes (*DEFA3*, *DEFA4* and *MPO*, **Figure 5C**) indicating that the presence of CD16^low^ LDN is responsible for the observed increased expression of genes associated with antimicrobial response.

### 3.4 Neutrophils from umbilical cord blood do not exert impaired activation of myeloperoxidase

**Figure 6:**
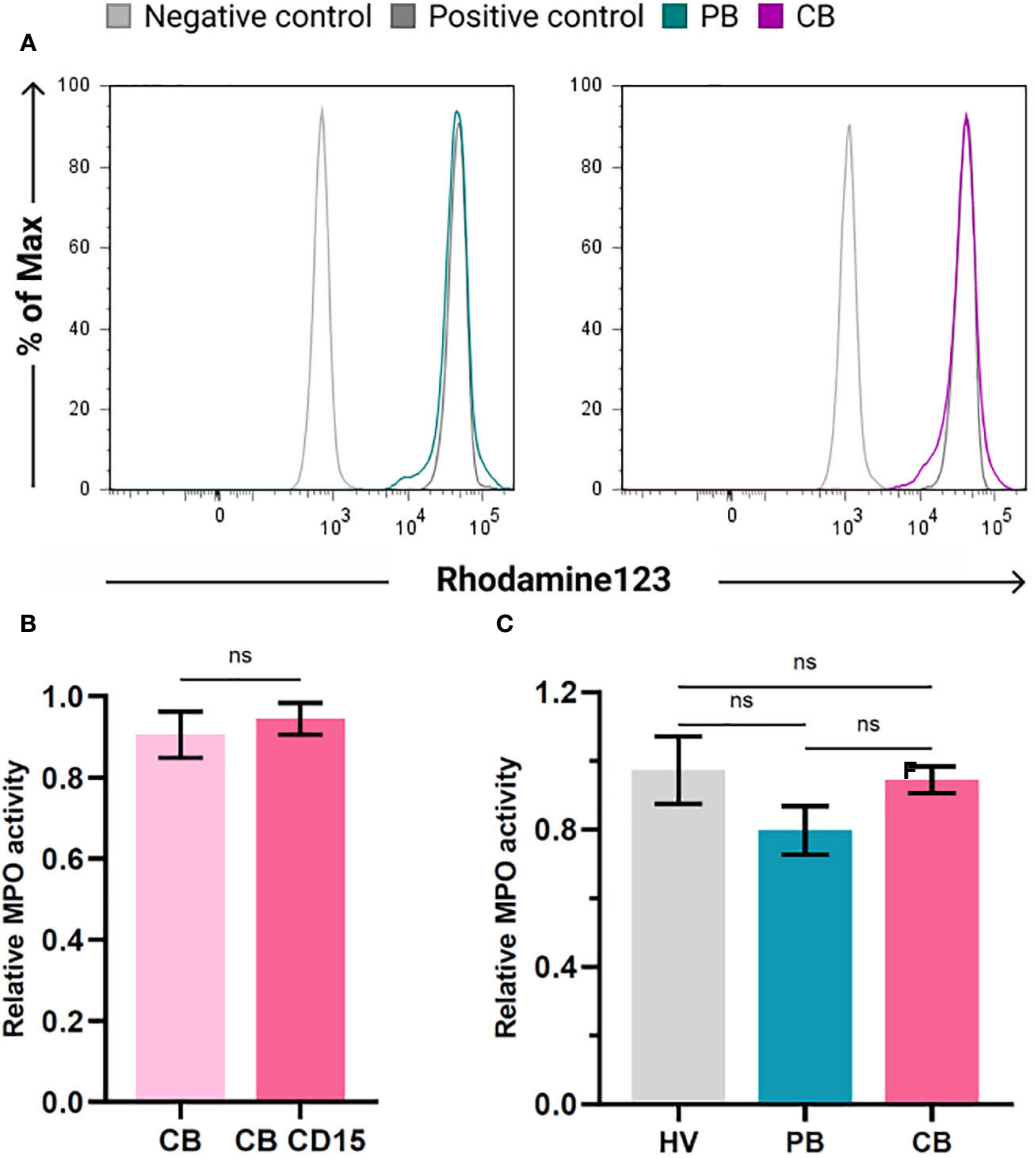
Activation of myeloperoxidase (MPO) in neutrophils of healthy volunteers (HV), peripheral blood (PB) and cord blood (CB). **(A)** Representative histograms documenting MPO activity in PB and CB using FagoFlowExKit. Oxidation of Dihydrorhodamine123 to fluorescent Rhodamine123 in cells stimulated with *E. coli* (green (PB) and violet (CB), nonstimulated neutrophils (grey line) and neutrophils stimulated with PMA (dark grey) - positive control. **(B)** Comparison of MPO activity in whole population of granulocytes and CD15+ granulocytes **(C)** comparison of relative MPO activity of neutrophils of HV, PB and CB. The data are shown as the mean ± SEM for the whole group. *ns p* > 0.05, two-tailed unpaired Student’s *t* test.

To elucidate whether the presence of CD16^low^ neutrophils affects the functional properties of neutrophils in CB, we studied their activation after stimulation with inflammatory stimulus *Escherichia coli*. To this end, we utilised FagoFlowEx Kit (EXBIO) employing dihydrorhodamine 123 oxidation to fluorescent rhodamine 123 by activated MPO (representative histograms shown at **Figure 6A**). Staining for CD15 employed for higher stringency of gating of neutrophil population did not exert any effect on their activation status (**Figure 6B**). As depicted in **Figure 6C**, the activation of neutrophils by inflammatory stimulus did not differ among the study groups.

## 4 Discussion

In this study, we analysed the relative proportion of granulocytes to other cell types in cord and peripheral blood. We found that the proportion of granulocytes to other cell types in CB, accounting for about 40% of all cells, was significantly lower compared to that of peripheral blood from mothers and healthy volunteers, where granulocytes account for about 70-80% of all cells. The lower frequency of granulocytes was accompanied by an increase in the absolute number of lymphocytes in CB, possibly accounting for the decreased proportion of granulocytes. Previous publications focusing on the relative representation of immune cell populations in CB described a similar increase in lymphocytes with immature phenotypical properties (57, 58).

Our study confirms prior observation by Weinhage et al. (41) that CB contains a high number of LDN, which they identified as CD15^+^CD16^low^CD64^high^ neutrophils with an immature phenotype. The origin of these immature cells is unclear since it was previously reported that CD16^low^CD62L^+^ LDN can be induced upon inflammatory stimulus (43) and during long-term chronic inflammation (25). The presence of inflammation during term labour (59, 60) may drive the expansion of the observed immature neutrophil populations in neonatal immune system.

Cord blood neutrophils have been previously reported to display impaired ability to release NETs (41) with CB CD15^+^CD16^low^CD64^high^ LDN exerting lower phagocytic capacity. In our results, the activation of CB neutrophils by *E. coli* was similar to neutrophils in PB and HV, indicating that CB neutrophils have lower phagocytic capacity but are still fully capable of activation and bacteria clearance. Previous studies have reported inconsistency in the impairment of neutrophil function in cord blood, with most studies pointing to greater impairment in neutrophils in preterm newborns (7). It has been suggested that neutrophils of term children do not display impaired intracellular functions, including generation of ROS, as opposed to children born preterm or with sepsis (61). However, they are unable to properly generate NETs (62). These findings suggest that neutrophils in term CB have an impaired ability to eliminate bacteria by NETosis but can efficiently phagocytize and kill infectious agents intracellularly.

It is important to mention that cord blood neutrophils can be influenced by the mother’s treatment before and during birth, e.g., by the application of anaesthetics (7, 63). While detailed studies on this theme are lacking, it would be interesting to elucidate whether the treatment of the mother has an impact on the composition and function of neutrophil populations. The myeloperoxidase activation assay used in this study is advantageous compared to previous studies (63, 64) as it does not require neutrophil isolation prior to the analysis, decreasing the risk of inadvertent neutrophil activation and loss of their ability to respond to stimuli.

We present a novel observation that CB neutrophils express higher levels of genes associated with antimicrobial function. This finding is consistent with previous studies reporting increased expression of *MPO*, *ELANE*, *DEFA4*, and *MMP8* genes in LDN isolated from SLE patients (32). The presented results suggest that LDN might be responsible for the observed increase in gene expression. We have to consider that MPO and other antimicrobial peptides tested in our study could be released during degranulation of LDN as described by Ssemaganda et al. (40). Possibly, elevated gene expression of antimicrobial peptides and MPO is a compensatory mechanism trying to renew the levels of these proteins. Mathias et al. (65) conducted a genome-wide expression profile of CB neutrophils from term neonates and found that unstimulated CB neutrophils produce higher levels of inflammatory cytokines such as IFN-γ, IL-1β, and TNF-α. Further, they showed that LPS treatment has a reduced capacity to upregulate genes linked to neutrophil activation, phagocytosis, and chemotaxis compared to adult peripheral blood neutrophils (65). This might suggest that CB neutrophils exert lower response to infectious agents to prevent harmful overt activation in the early stages after birth. This effect may be dependent on neutrophil composition since the population frequencies are different at birth (66). Neutrophil numbers normalize to adult levels by about four weeks of age (67).

Jimenez et al. (35) discovered that the accumulation of C-reactive protein (CRP) triggers the release of MDSC from mouse bone marrow, leading to an increase in ROS production and suppression of T cell proliferation. Similar effect was also seen in isolated human neutrophils. While CRP is commonly used as a biomarker for acute inflammation, it is not as sensitive as neutrophil CD64 expression when diagnosing neonatal sepsis (68). Injarabian et al. (69) addressed the effect of metabolic pathways and microenvironment on neutrophil differentiation and phenotype, with the metabolic shift during inflammation potentially leading to the formation of LDN. Our study found that CD15^+^CD16^low^CD64^high^ and CD15^+^CD16^low^CD62L^+^ neutrophils were enriched in the low-density compartment of CBMC. The high expression of CD64 on immature CD16^low^ neutrophil subpopulation in CB and their further recruitment during infection could contribute to the high sepsis rates observed in neonates.

Gene expression analysis revealed that CB neutrophils displayed increased expression of genes related to antimicrobial functions compared to maternal PB and PB of healthy volunteers. Maternal PB neutrophils exhibited slightly higher expression of several genes, potentially facilitating neutrophils’ role in dissolving fetal membranes via the production of MMPs during labour (70). Both CB and maternal PB neutrophils displayed elevated levels of the *Arg1* gene, which is, along with IL-10 and PD-L1, associated with suppressive function of PMN-MDSC (71, 72). PD-L1 expression was found to be associated with immune suppressive mechanisms in pregnancy, and its upregulation was observed in maternal PB neutrophils, suggesting regulatory mechanisms that differ from those in CB. The mode of delivery was found to affect neutrophil gene expression, with children delivered by CS showing increased expression of genes related to antimicrobial response, while children delivered by VD displayed increased expression of suppressive molecules.

## 5 Conclusions

Our study highlights a significant aspect of the complex neonatal immune system, particularly the role of umbilical cord blood neutrophils. It confirms the presence of CD16^low^ neutrophil subpopulation in cord blood, exhibiting an immature phenotype. CD16^low^ neutrophil subpopulation plays a crucial role in the heightened expression of genes associated with antimicrobial responses, including myeloperoxidase, defensins, and neutrophil elastase. Interestingly, cord blood neutrophils demonstrate proper activation and oxidative burst when challenged with an inflammatory stimulus compared to the peripheral blood neutrophils of adults.

Importantly, our findings suggest that the mode of delivery can influence neonatal immune properties. Although we recognize the limitations of our study, we hope that the results presented here will provide valuable insights into the immune landscape of newborns, emphasizing the importance of distinct neutrophil subpopulations in neonatal immunity and their potential impact on early postnatal health. Further research into these immune mechanisms may help develop effective neonatal care and infection prevention strategies.

## Data availability statement

The original contributions presented in the study are included in the article/**Supplementary Materials**, further inquiries can be directed to the corresponding author/s.

## Ethics statement

The studies involving humans were approved by Ethical Committee of Institute for the Care of Mother and Child. The studies were conducted in accordance with the local legislation and institutional requirements. Written informed consent for participation in this study was provided by the participants’ legal guardians/next of kin.

## Author contributions

EM: Methodology, Investigation, Formal analysis, Writing – review & editing, Writing – original draft, Conceptualization. VČ: Writing – review & editing, Methodology, Investigation. ON: Methodology, Investigation, Writing – review & editing. PP: Writing – review & editing, Methodology, Investigation. KB: Project administration, Writing – review & editing, Investigation. ZH: Supervision, Funding acquisition, Writing – review & editing, Writing – original draft. JH: Resources, Conceptualization, Writing – review & editing, Writing – original draft, Supervision, Funding acquisition.
