# Supplementary material for: Immature neutrophils in cord blood exert increased expression of genes associated with antimicrobial function
Source: Front Immunol. 2024 Mar 26;15:1368624. doi: 10.3389/fimmu.2024.1368624 (PMC11002259; doi:10.3389/fimmu.2024.1368624)
Supplement: Supplementary file 1 [file DataSheet_1.pdf]

Supplementary Material

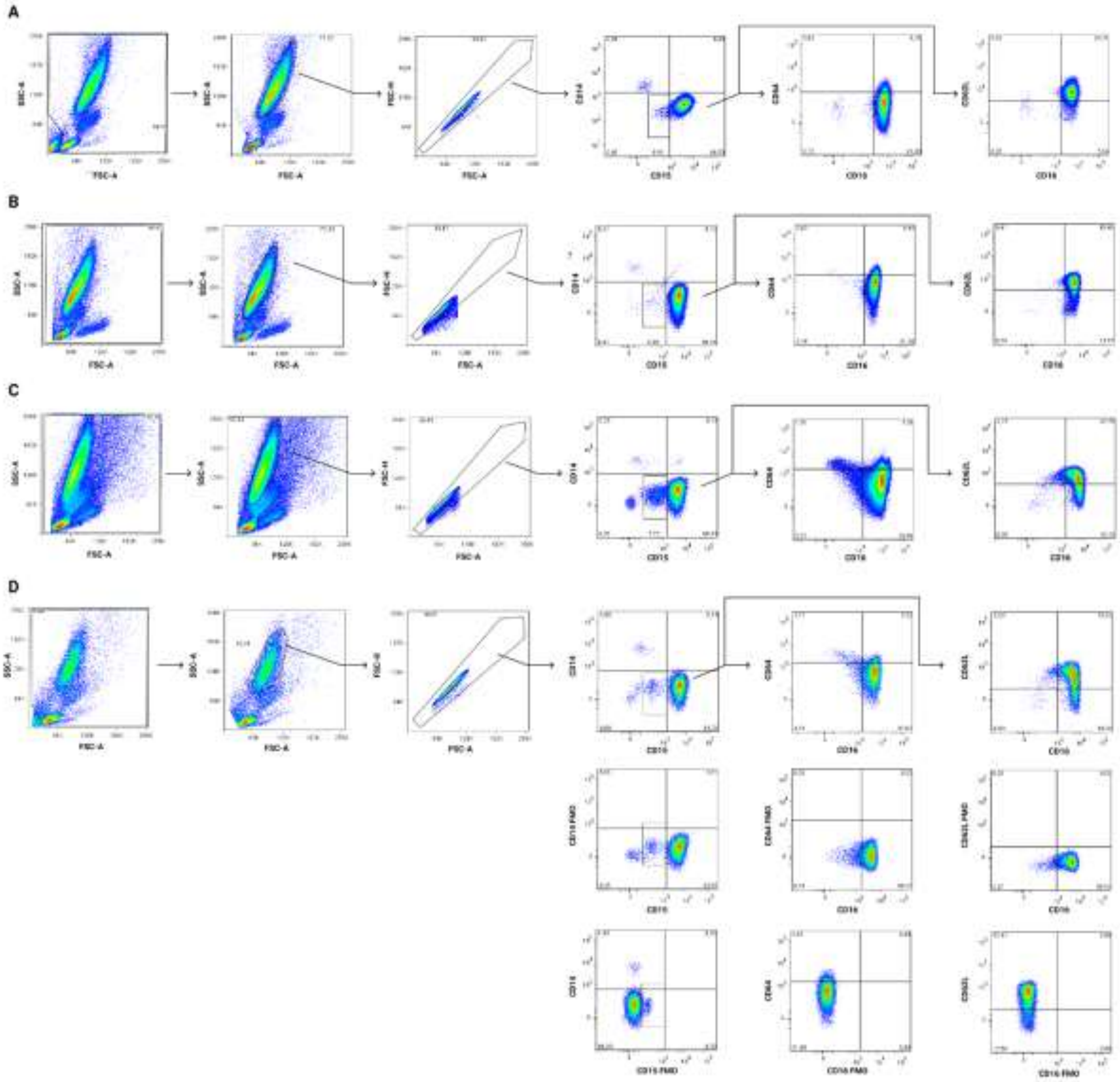

Supplementary Figure 1. Gating strategy for the identification of neutrophil subpopulations in peripheral blood of healthy volunteers (A), mothers (B), and in cord blood (C). Fluorescence Minus One (FMO) controls for individual markers shown on a representative CB sample (D).

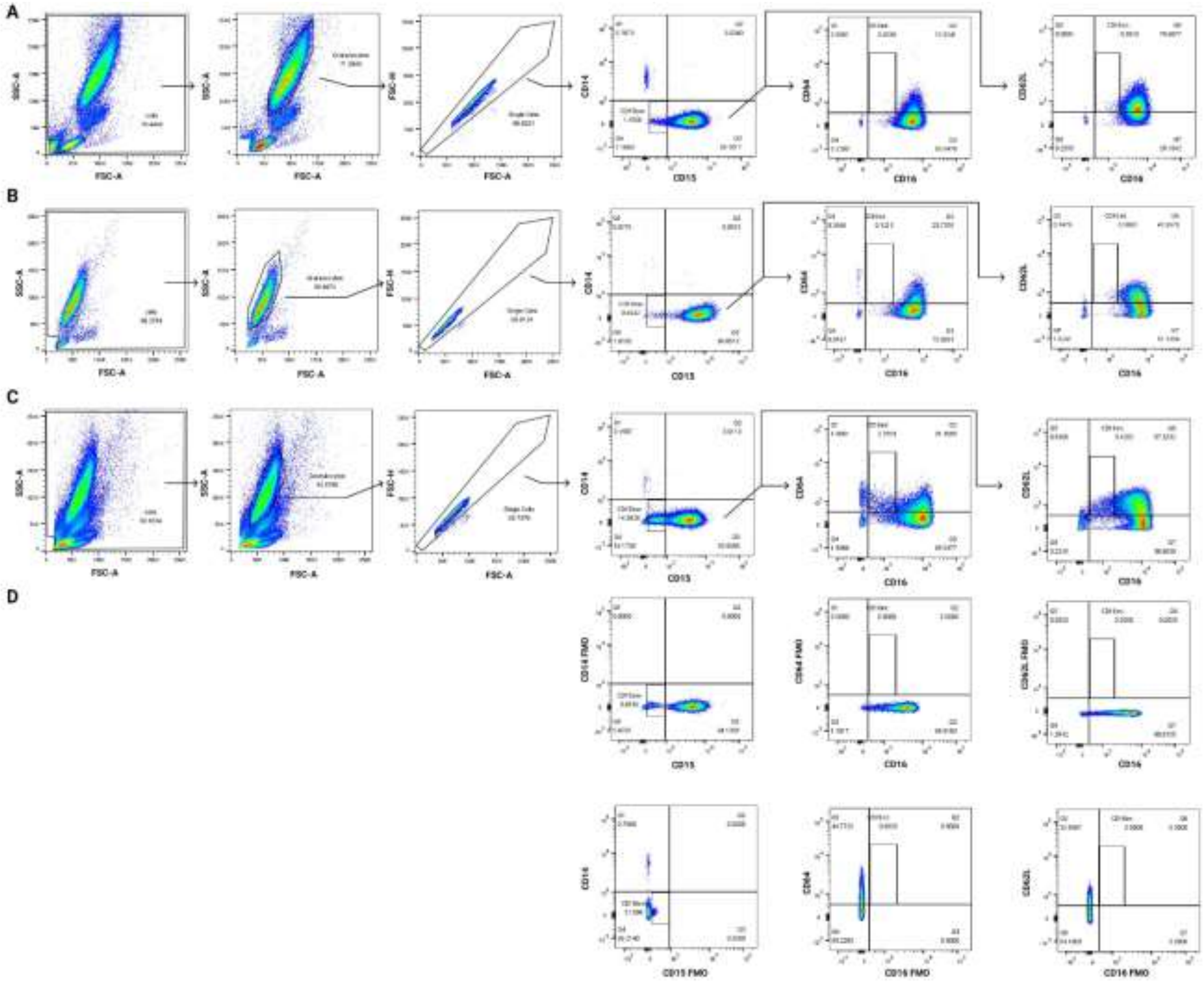

Supplementary Figure 2. Gating strategy for the identification of neutrophil subpopulations in peripheral blood of healthy volunteers (A), mothers (B), and in cord blood (C) with detailed identification of distinct subpopulations based on cell surface presence of CD16. Gates are set strictly according to Fluorescence Minus One (FMO) controls. FMO for individual markers shown on a representative CB sample (D).

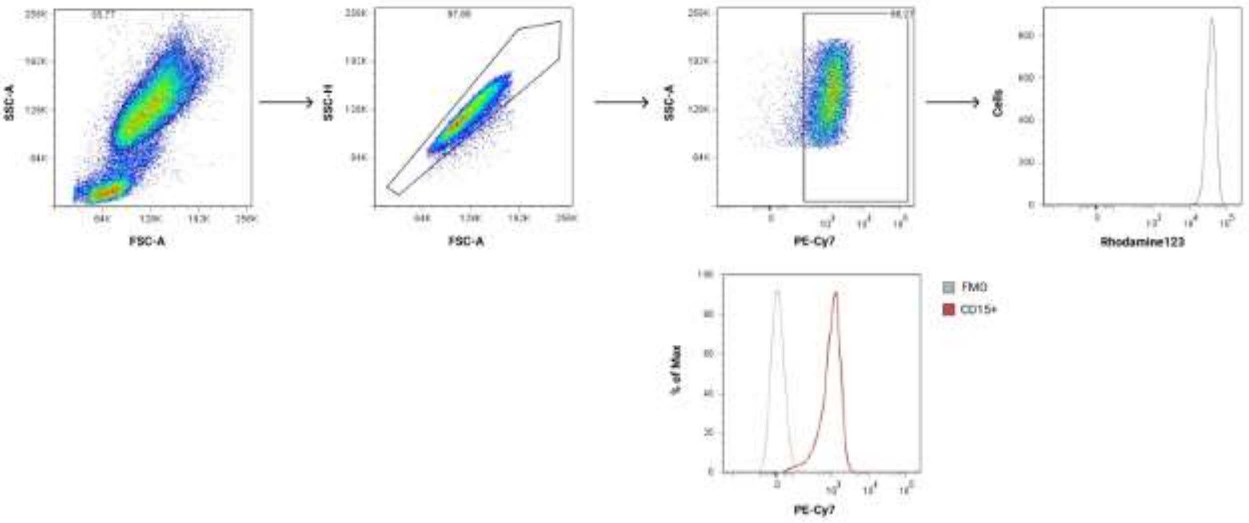

Supplementary Figure 3. Gating strategy used to quantify the relative myeloperoxidase (MPO) activity using the FagoFlowExKit. Representative gating of the positive control from cord blood sample is shown (cells stimulated with PMA).

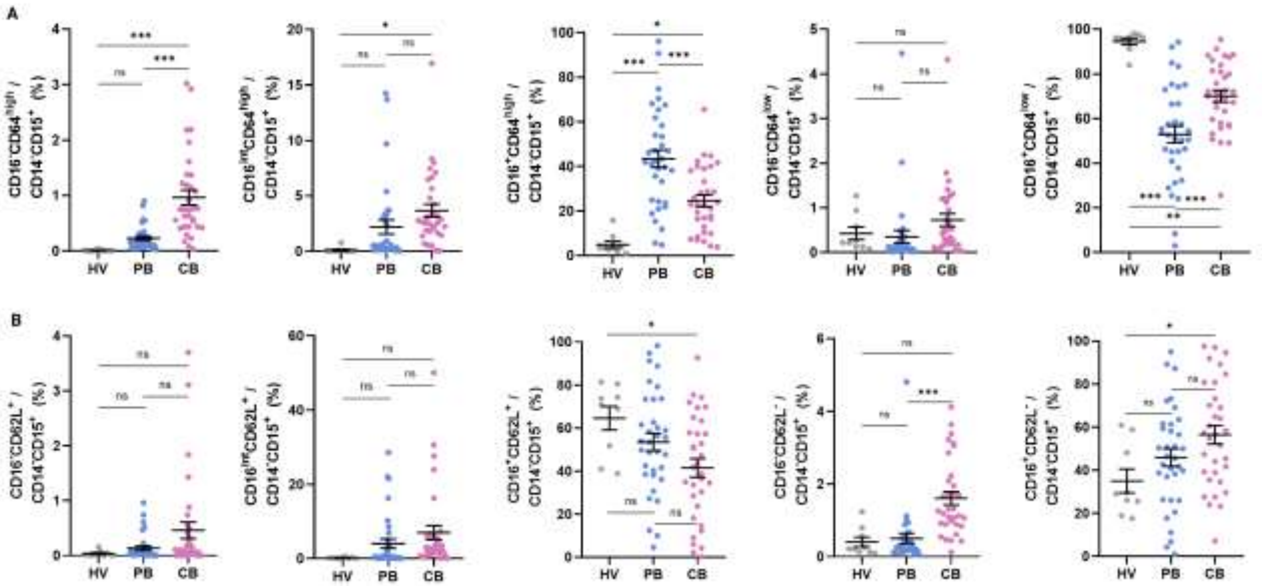

Supplementary Figure 4. Proportion of particular neutrophil subset determined by flow cytometry according to cell surface presence of CD16 as documented in Supplementary Figure 2. (A) Proportion of $CD16^{-}CD64^{high}$, $CD16^{int}CD64^{high}$, $CD16^{+}CD64^{high}$, $CD16^{-}CD64^{low}$ and $CD16^{+}CD64^{low}$ neutrophils within $CD14^{-}CD15^{+}$ subset. (B) Proportion of $CD16^{-}CD62L^{+}$, $CD16^{int}CD62L^{+}$, $CD16^{+}CD62L^{+}$, $CD16^{-}CD62L^{-}$ and $CD16^{+}CD62L^{-}$ neutrophils within $CD14^{-}CD15^{+}$ subset.

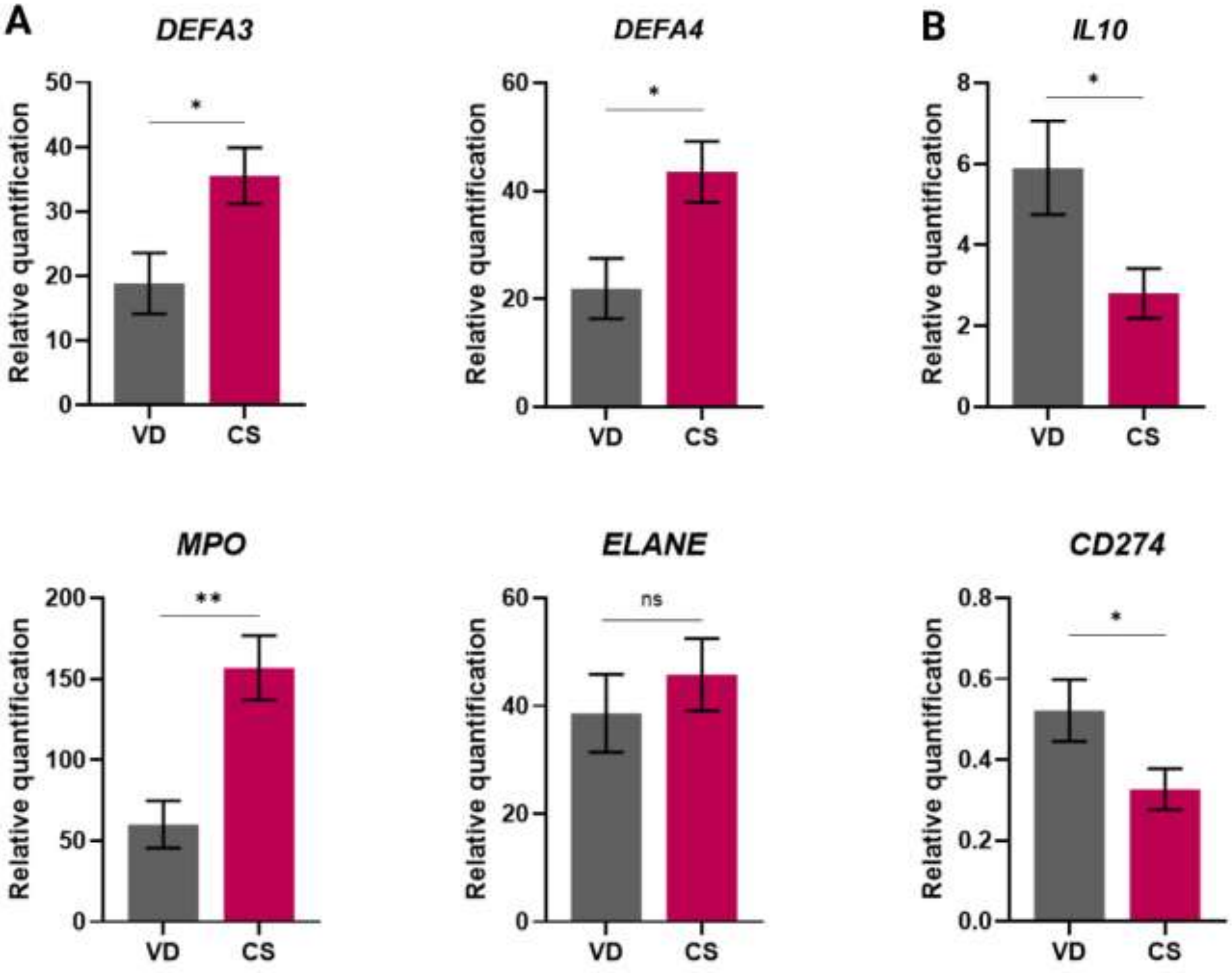

Supplementary Figure 5. Gene expression analysis of neutrophils isolated from umbilical cord blood of children delivered by vaginal delivery (VD) or caesarean section (CS). (A) alpha defensin 3, alpha defensin 4, myeloperoxidase, neutrophil elastase (VD: n = 16, CS: n = 38). (B) Interleukin-10, Programmed death-ligand 1 (PD-L1/CD274). The data are shown as the mean ± SEM for the whole group. Asterisks indicate $*p < 0.05$, $**p < 0.01$, two-tailed unpaired Student's *t* test.
